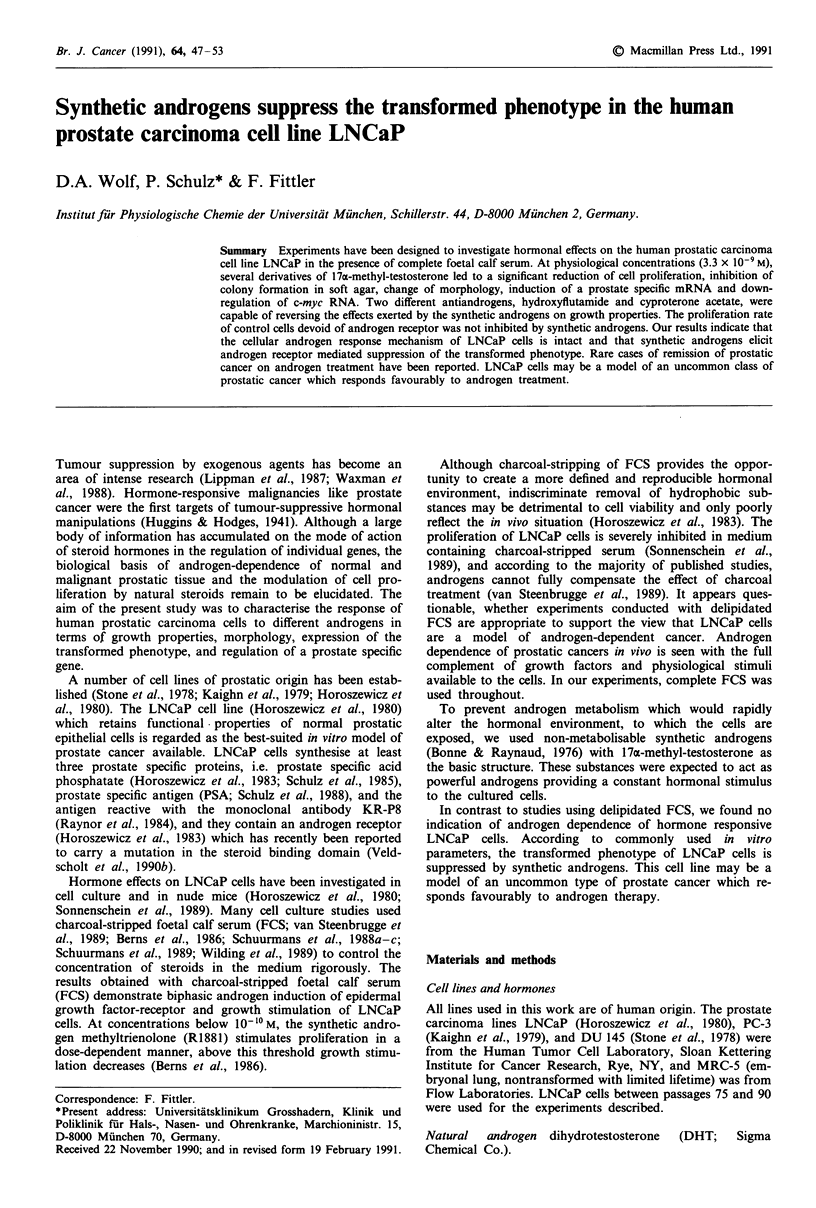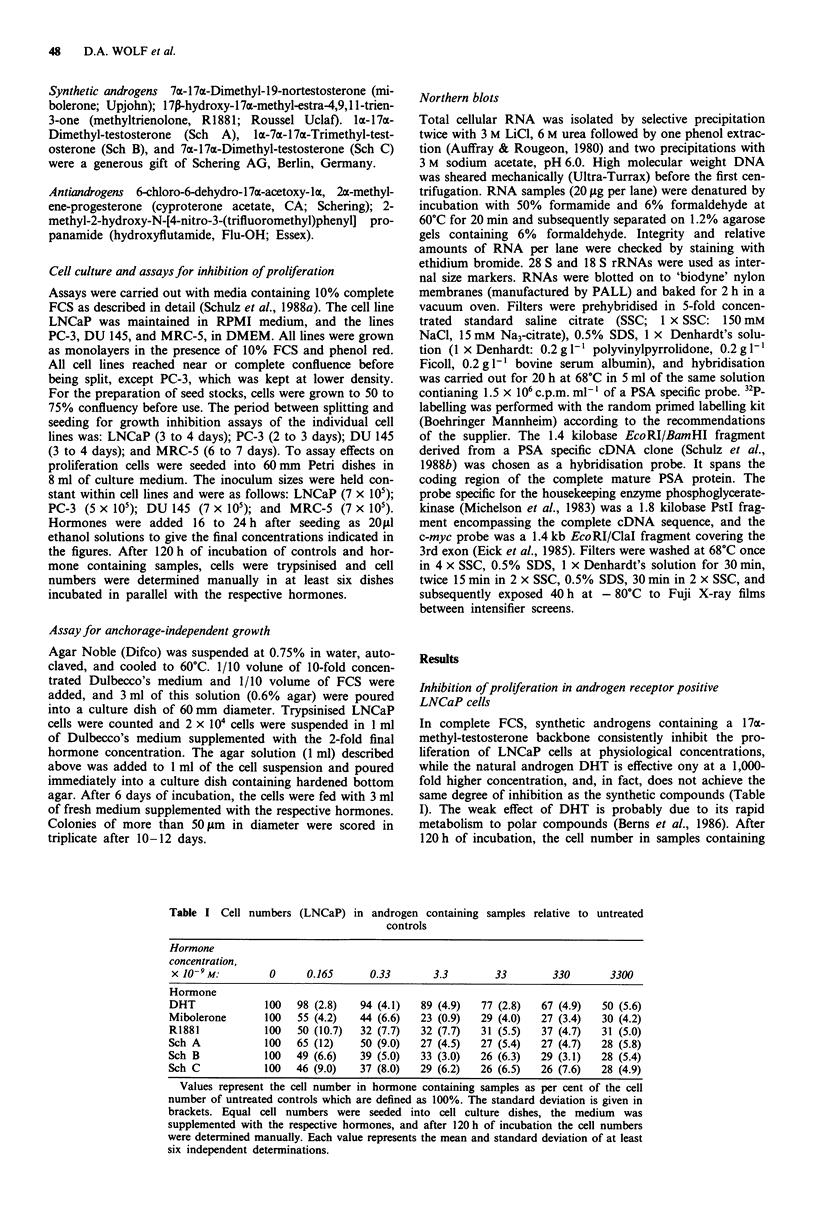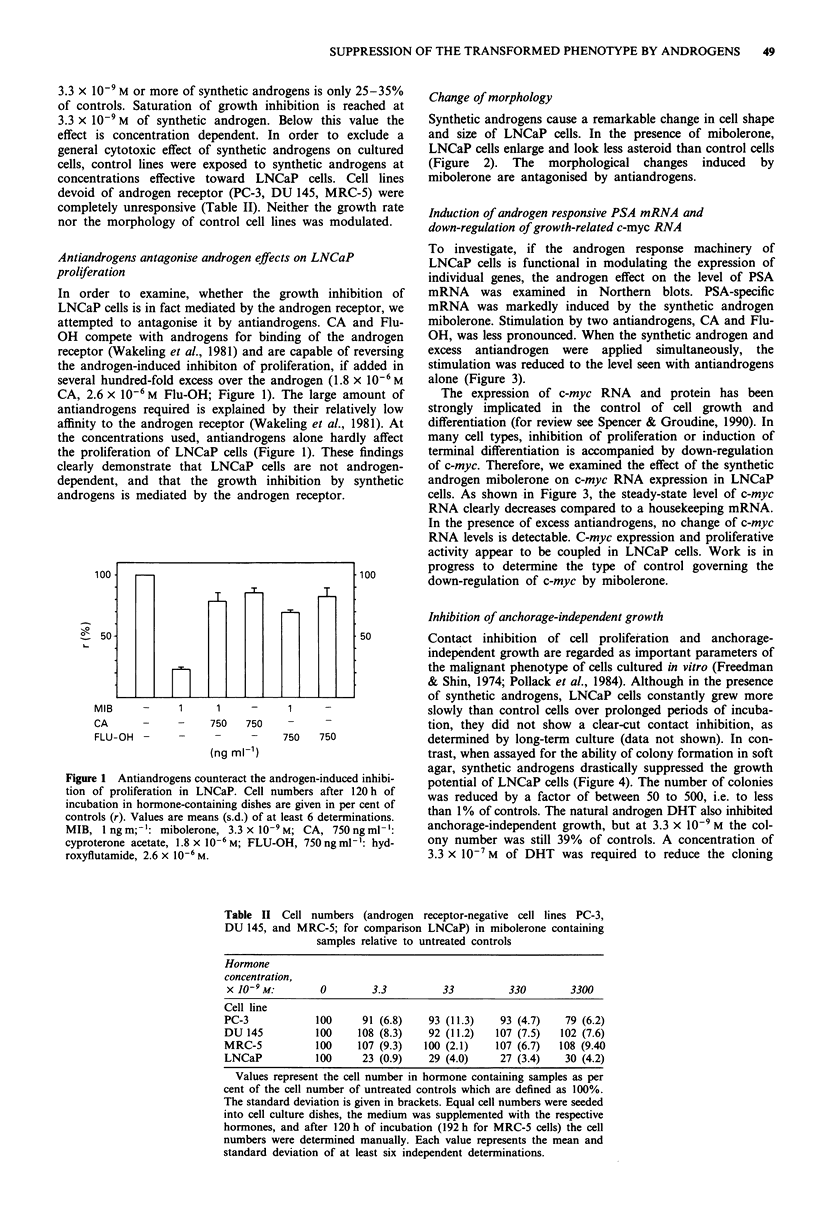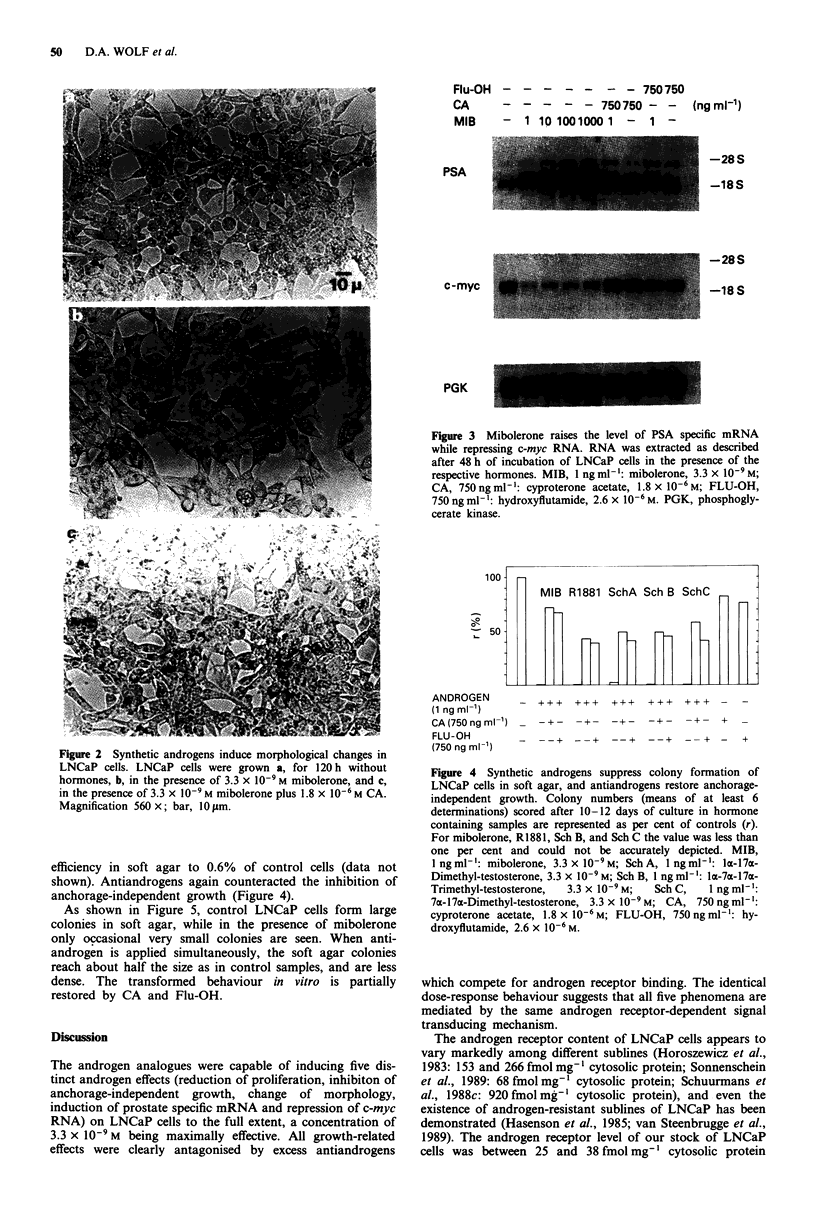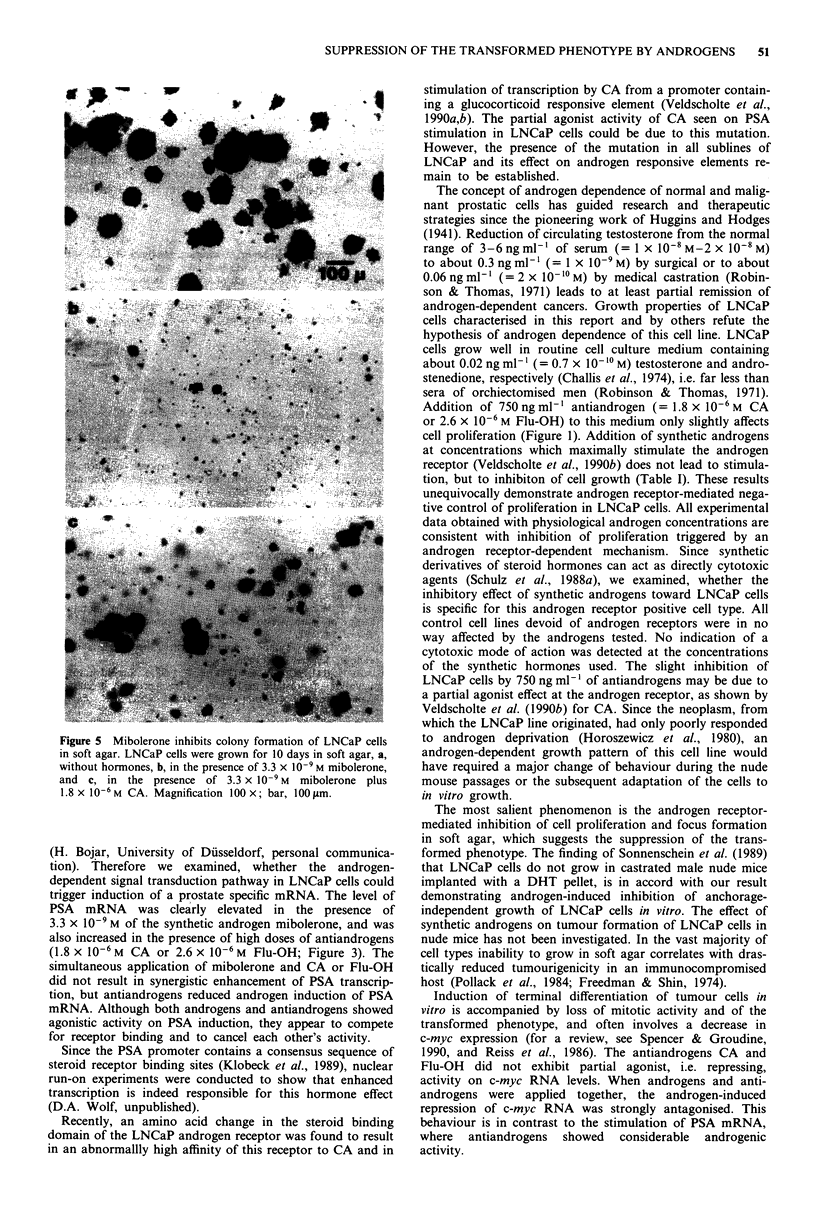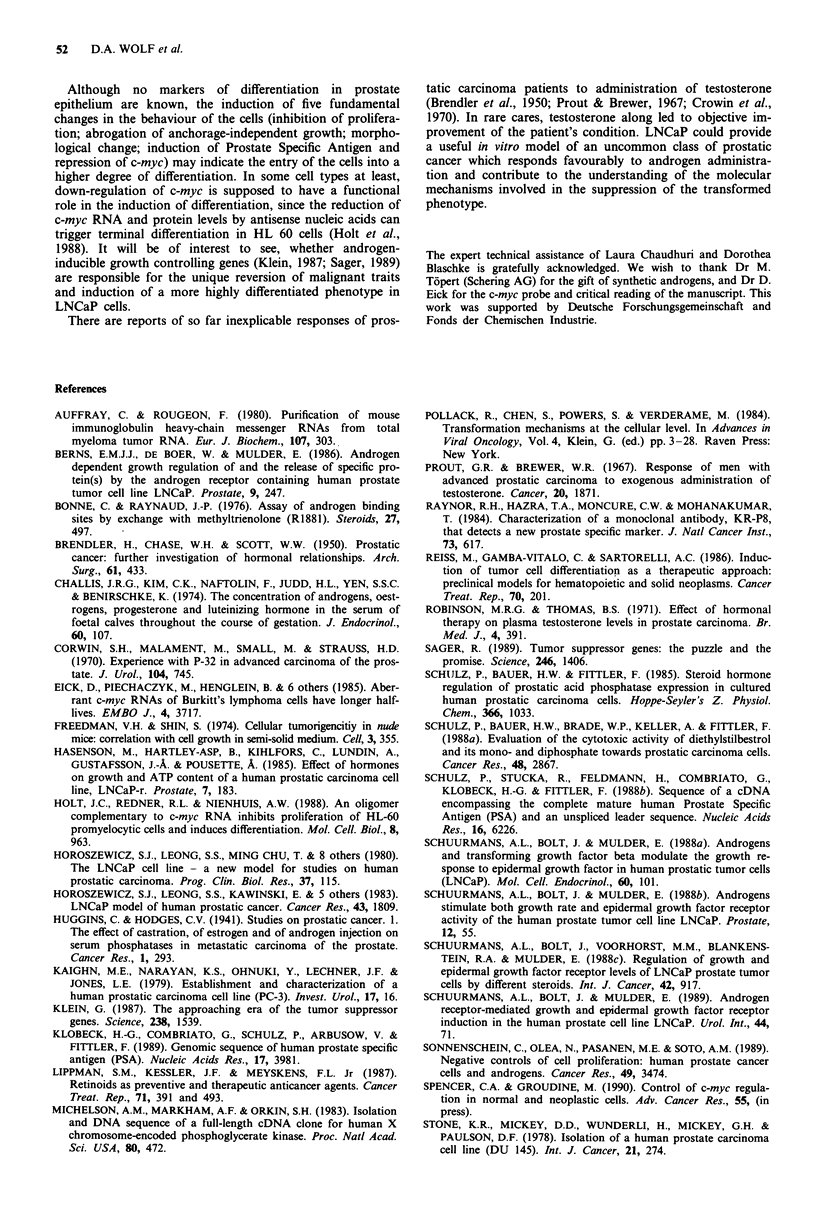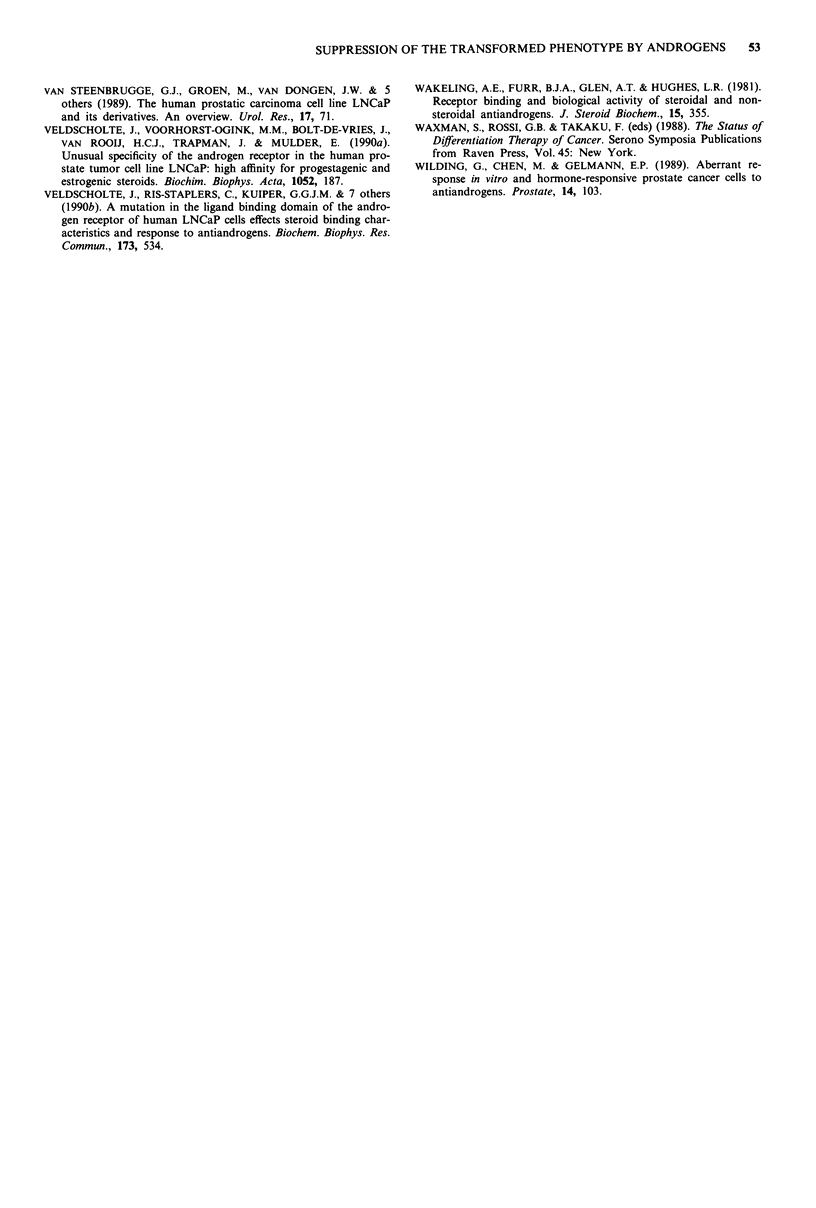# Synthetic androgens suppress the transformed phenotype in the human prostate carcinoma cell line LNCaP.

**DOI:** 10.1038/bjc.1991.237

**Published:** 1991-07

**Authors:** D. A. Wolf, P. Schulz, F. Fittler

**Affiliations:** Institut für Physiologische Chemie, Universität München, Germany.

## Abstract

**Images:**


*Br. J. Cancer* (1991), **64**, 47–53 © Macmillan Press Ltd., 1991

### Synthetic androgens suppress the transformed phenotype in the human prostate carcinoma cell line LNCaP

D.A. Wolf, P. Schulz* & F. Fittler

*Institut für Physiologische Chemie der Universität München, Schillerstr. 44, D-8000 München 2, Germany.*

**Summary** Experiments have been designed to investigate hormonal effects on the human prostatic carcinoma cell line LNCaP in the presence of complete foetal calf serum. At physiological concentrations ($3.3 \times 10^{-9}$ M), several derivatives of 17α-methyl-testosterone led to a significant reduction of cell proliferation, inhibition of colony formation in soft agar, change of morphology, induction of a prostate specific mRNA and down-regulation of c-*myc* RNA. Two different antiandrogens, hydroxyflutamide and cyproterone acetate, were capable of reversing the effects exerted by the synthetic androgens on growth properties. The proliferation rate of control cells devoid of androgen receptor was not inhibited by synthetic androgens. Our results indicate that the cellular androgen response mechanism of LNCaP cells is intact and that synthetic androgens elicit androgen receptor mediated suppression of the transformed phenotype. Rare cases of remission of prostatic cancer on androgen treatment have been reported. LNCaP cells may be a model of an uncommon class of prostatic cancer which responds favourably to androgen treatment.

Tumour suppression by exogenous agents has become an area of intense research (Lippman *et al.*, 1987; Waxman *et al.*, 1988). Hormone-responsive malignancies like prostate cancer were the first targets of tumour-suppressive hormonal manipulations (Huggins & Hodges, 1941). Although a large body of information has accumulated on the mode of action of steroid hormones in the regulation of individual genes, the biological basis of androgen-dependence of normal and malignant prostatic tissue and the modulation of cell proliferation by natural steroids remain to be elucidated. The aim of the present study was to characterise the response of human prostatic carcinoma cells to different androgens in terms of growth properties, morphology, expression of the transformed phenotype, and regulation of a prostate specific gene.

A number of cell lines of prostatic origin has been established (Stone *et al.*, 1978; Kaighn *et al.*, 1979; Horoszewicz *et al.*, 1980). The LNCaP cell line (Horoszewicz *et al.*, 1980) which retains functional properties of normal prostatic epithelial cells is regarded as the best-suited *in vitro* model of prostate cancer available. LNCaP cells synthesise at least three prostate specific proteins, i.e. prostate specific acid phosphatate (Horoszewicz *et al.*, 1983; Schulz *et al.*, 1985), prostate specific antigen (PSA; Schulz *et al.*, 1988), and the antigen reactive with the monoclonal antibody KR-P8 (Raynor *et al.*, 1984), and they contain an androgen receptor (Horoszewicz *et al.*, 1983) which has recently been reported to carry a mutation in the steroid binding domain (Veldscholt *et al.*, 1990*b*).

Hormone effects on LNCaP cells have been investigated in cell culture and in nude mice (Horoszewicz *et al.*, 1980; Sonnenschein *et al.*, 1989). Many cell culture studies used charcoal-stripped foetal calf serum (FCS; van Steenbrugge *et al.*, 1989; Berns *et al.*, 1986; Schuurmans *et al.*, 1988*a–c*; Schuurmans *et al.*, 1989; Wilding *et al.*, 1989) to control the concentration of steroids in the medium rigorously. The results obtained with charcoal-stripped foetal calf serum (FCS) demonstrate biphasic androgen induction of epidermal growth factor-receptor and growth stimulation of LNCaP cells. At concentrations below $10^{-10}$ M, the synthetic androgen methyltrienolone (R1881) stimulates proliferation in a dose-dependent manner, above this threshold growth stimulation decreases (Berns *et al.*, 1986).

Correspondence: F. Fittler.
*Present address: Universitätsklinikum Grosshadern, Klinik und Poliklinik für Hals-, Nasen- und Ohrenkranke, Marchioninistr. 15, D-8000 München 70, Germany.
Received 22 November 1990; and in revised form 19 February 1991.

Although charcoal-stripping of FCS provides the opportunity to create a more defined and reproducible hormonal environment, indiscriminate removal of hydrophobic substances may be detrimental to cell viability and only poorly reflect the *in vivo* situation (Horoszewicz *et al.*, 1983). The proliferation of LNCaP cells is severely inhibited in medium containing charcoal-stripped serum (Sonnenschein *et al.*, 1989), and according to the majority of published studies, androgens cannot fully compensate the effect of charcoal treatment (van Steenbrugge *et al.*, 1989). It appears questionable, whether experiments conducted with delipidated FCS are appropriate to support the view that LNCaP cells are a model of androgen-dependent cancer. Androgen dependence of prostatic cancers *in vivo* is seen with the full complement of growth factors and physiological stimuli available to the cells. In our experiments, complete FCS was used throughout.

To prevent androgen metabolism which would rapidly alter the hormonal environment, to which the cells are exposed, we used non-metabolisable synthetic androgens (Bonne & Raynaud, 1976) with 17α-methyl-testosterone as the basic structure. These substances were expected to act as powerful androgens providing a constant hormonal stimulus to the cultured cells.

In contrast to studies using delipidated FCS, we found no indication of androgen dependence of hormone responsive LNCaP cells. According to commonly used *in vitro* parameters, the transformed phenotype of LNCaP cells is suppressed by synthetic androgens. This cell line may be a model of an uncommon type of prostate cancer which responds favourably to androgen therapy.

### Materials and methods

### *Cell lines and hormones*

All lines used in this work are of human origin. The prostate carcinoma lines LNCaP (Horoszewicz *et al.*, 1980), PC-3 (Kaighn *et al.*, 1979), and DU 145 (Stone *et al.*, 1978) were from the Human Tumor Cell Laboratory, Sloan Kettering Institute for Cancer Research, Rye, NY, and MRC-5 (embryonal lung, nontransformed with limited lifetime) was from Flow Laboratories. LNCaP cells between passages 75 and 90 were used for the experiments described.

*Natural androgen* dihydrotestosterone (DHT; Sigma Chemical Co.).

*Synthetic androgens* 7α-17α-Dimethyl-19-nortestosterone (mibolerone; Upjohn); 17β-hydroxy-17α-methyl-estra-4,9,11-trien-3-one (methyltrienolone, R1881; Roussel Uclaf). 1α-17α-Dimethyl-testosterone (Sch A), 1α-7α-17α-Trimethyl-testosterone (Sch B), and 7α-17α-Dimethyl-testosterone (Sch C) were a generous gift of Schering AG, Berlin, Germany.

*Antiandrogens* 6-chloro-6-dehydro-17α-acetoxy-1α, 2α-methylene-progesterone (cyproterone acetate, CA; Schering); 2-methyl-2-hydroxy-N-[4-nitro-3-(trifluoromethyl)phenyl] propanamide (hydroxyflutamide, Flu-OH; Essex).

### *Cell culture and assays for inhibition of proliferation*

Assays were carried out with media containing 10% complete FCS as described in detail (Schulz *et al.*, 1988*a*). The cell line LNCaP was maintained in RPMI medium, and the lines PC-3, DU 145, and MRC-5, in DMEM. All lines were grown as monolayers in the presence of 10% FCS and phenol red. All cell lines reached near or complete confluence before being split, except PC-3, which was kept at lower density. For the preparation of seed stocks, cells were grown to 50 to 75% confluency before use. The period between splitting and seeding for growth inhibition assays of the individual cell lines was: LNCaP (3 to 4 days); PC-3 (2 to 3 days); DU 145 (3 to 4 days); and MRC-5 (6 to 7 days). To assay effects on proliferation cells were seeded into 60 mm Petri dishes in 8 ml of culture medium. The inoculum sizes were held constant within cell lines and were as follows: LNCaP ($7 \times 10^5$); PC-3 ($5 \times 10^5$); DU 145 ($7 \times 10^5$); and MRC-5 ($7 \times 10^5$). Hormones were added 16 to 24 h after seeding as 20 μl ethanol solutions to give the final concentrations indicated in the figures. After 120 h of incubation of controls and hormone containing samples, cells were trypsinised and cell numbers were determined manually in at least six dishes incubated in parallel with the respective hormones.

### *Assay for anchorage-independent growth*

Agar Noble (Difco) was suspended at 0.75% in water, autoclaved, and cooled to 60°C. 1/10 volume of 10-fold concentrated Dulbecco's medium and 1/10 volume of FCS were added, and 3 ml of this solution (0.6% agar) were poured into a culture dish of 60 mm diameter. Trypsinised LNCaP cells were counted and $2 \times 10^4$ cells were suspended in 1 ml of Dulbecco's medium supplemented with the 2-fold final hormone concentration. The agar solution (1 ml) described above was added to 1 ml of the cell suspension and poured immediately into a culture dish containing hardened bottom agar. After 6 days of incubation, the cells were fed with 3 ml of fresh medium supplemented with the respective hormones. Colonies of more than 50 μm in diameter were scored in triplicate after 10–12 days.

### *Northern blots*

Total cellular RNA was isolated by selective precipitation twice with 3 M LiCl, 6 M urea followed by one phenol extraction (Auffray & Rougeon, 1980) and two precipitations with 3 M sodium acetate, pH 6.0. High molecular weight DNA was sheared mechanically (Ultra-Turrax) before the first centrifugation. RNA samples (20 μg per lane) were denatured by incubation with 50% formamide and 6% formaldehyde at 60°C for 20 min and subsequently separated on 1.2% agarose gels containing 6% formaldehyde. Integrity and relative amounts of RNA per lane were checked by staining with ethidium bromide. 28 S and 18 S rRNAs were used as internal size markers. RNAs were blotted on to 'biodyne' nylon membranes (manufactured by PALL) and baked for 2 h in a vacuum oven. Filters were prehybridised in 5-fold concentrated standard saline citrate (SSC; 1 × SSC: 150 mM NaCl, 15 mM $Na_3$-citrate), 0.5% SDS, 1 × Denhardt's solution (1 × Denhardt: 0.2 g $l^{-1}$ polyvinylpyrrolidone, 0.2 g $l^{-1}$ Ficoll, 0.2 g $l^{-1}$ bovine serum albumin), and hybridisation was carried out for 20 h at 68°C in 5 ml of the same solution contianing $1.5 \times 10^6$ c.p.m. $ml^{-1}$ of a PSA specific probe. $^{32}P$-labelling was performed with the random primed labelling kit (Boehringer Mannheim) according to the recommendations of the supplier. The 1.4 kilobase *Eco*RI/*Bam*HI fragment derived from a PSA specific cDNA clone (Schulz *et al.*, 1988*b*) was chosen as a hybridisation probe. It spans the coding region of the complete mature PSA protein. The probe specific for the housekeeping enzyme phosphoglycerate-kinase (Michelson *et al.*, 1983) was a 1.8 kilobase PstI fragment encompassing the complete cDNA sequence, and the c-*myc* probe was a 1.4 kb *Eco*RI/ClaI fragment covering the 3rd exon (Eick *et al.*, 1985). Filters were washed at 68°C once in 4 × SSC, 0.5% SDS, 1 × Denhardt's solution for 30 min, twice 15 min in 2 × SSC, 0.5% SDS, 30 min in 2 × SSC, and subsequently exposed 40 h at − 80°C to Fuji X-ray films between intensifier screens.

### Results

### *Inhibition of proliferation in androgen receptor positive LNCaP cells*

In complete FCS, synthetic androgens containing a 17α-methyl-testosterone backbone consistently inhibit the proliferation of LNCaP cells at physiological concentrations, while the natural androgen DHT is effective ony at a 1,000-fold higher concentration, and, in fact, does not achieve the same degree of inhibition as the synthetic compounds (Table I). The weak effect of DHT is probably due to its rapid metabolism to polar compounds (Berns *et al.*, 1986). After 120 h of incubation, the cell number in samples containing

**Table I** Cell numbers (LNCaP) in androgen containing samples relative to untreated controls

| *Hormone concentration, $\times 10^{-9}$ M:* | *0* | *0.165* | *0.33* | *3.3* | *33* | *330* | *3300* |
|---|---|---|---|---|---|---|---|
| Hormone | | | | | | | |
| DHT | 100 | 98 (2.8) | 94 (4.1) | 89 (4.9) | 77 (2.8) | 67 (4.9) | 50 (5.6) |
| Mibolerone | 100 | 55 (4.2) | 44 (6.6) | 23 (0.9) | 29 (4.0) | 27 (3.4) | 30 (4.2) |
| R1881 | 100 | 50 (10.7) | 32 (7.7) | 32 (7.7) | 31 (5.5) | 37 (4.7) | 31 (5.0) |
| Sch A | 100 | 65 (12) | 50 (9.0) | 27 (4.5) | 27 (5.4) | 27 (4.7) | 28 (5.8) |
| Sch B | 100 | 49 (6.6) | 39 (5.0) | 33 (3.0) | 26 (6.3) | 29 (3.1) | 28 (5.4) |
| Sch C | 100 | 46 (9.0) | 37 (8.0) | 29 (6.2) | 26 (6.5) | 26 (7.6) | 28 (4.9) |

Values represent the cell number in hormone containing samples as per cent of the cell number of untreated controls which are defined as 100%. The standard deviation is given in brackets. Equal cell numbers were seeded into cell culture dishes, the medium was supplemented with the respective hormones, and after 120 h of incubation the cell numbers were determined manually. Each value represents the mean and standard deviation of at least six independent determinations.

$3.3 \times 10^{-9}$ M or more of synthetic androgens is only 25–35% of controls. Saturation of growth inhibition is reached at $3.3 \times 10^{-9}$ M of synthetic androgen. Below this value the effect is concentration dependent. In order to exclude a general cytotoxic effect of synthetic androgens on cultured cells, control lines were exposed to synthetic androgens at concentrations effective toward LNCaP cells. Cell lines devoid of androgen receptor (PC-3, DU 145, MRC-5) were completely unresponsive (Table II). Neither the growth rate nor the morphology of control cell lines was modulated.

### *Antiandrogens antagonise androgen effects on LNCaP proliferation*

In order to examine, whether the growth inhibition of LNCaP cells is in fact mediated by the androgen receptor, we attempted to antagonise it by antiandrogens. CA and Flu-OH compete with androgens for binding of the androgen receptor (Wakeling *et al.*, 1981) and are capable of reversing the androgen-induced inhibiton of proliferation, if added in several hundred-fold excess over the androgen ($1.8 \times 10^{-6}$ M CA, $2.6 \times 10^{-6}$ M Flu-OH; Figure 1). The large amount of antiandrogens required is explained by their relatively low affinity to the androgen receptor (Wakeling *et al.*, 1981). At the concentrations used, antiandrogens alone hardly affect the proliferation of LNCaP cells (Figure 1). These findings clearly demonstrate that LNCaP cells are not androgen-dependent, and that the growth inhibition by synthetic androgens is mediated by the androgen receptor.

**Figure 1** Antiandrogens counteract the androgen-induced inhibition of proliferation in LNCaP. Cell numbers after 120 h of incubation in hormone-containing dishes are given in per cent of controls ($r$). Values are means (s.d.) of at least 6 determinations. MIB, 1 ng m;$^{-1}$: mibolerone, $3.3 \times 10^{-9}$ M; CA, 750 ng ml$^{-1}$: cyproterone acetate, $1.8 \times 10^{-6}$ M; FLU-OH, 750 ng ml$^{-1}$: hydroxyflutamide, $2.6 \times 10^{-6}$ M.

### *Change of morphology*

Synthetic androgens cause a remarkable change in cell shape and size of LNCaP cells. In the presence of mibolerone, LNCaP cells enlarge and look less asteroid than control cells (Figure 2). The morphological changes induced by mibolerone are antagonised by antiandrogens.

### *Induction of androgen responsive PSA mRNA and down-regulation of growth-related c-*myc *RNA*

To investigate, if the androgen response machinery of LNCaP cells is functional in modulating the expression of individual genes, the androgen effect on the level of PSA mRNA was examined in Northern blots. PSA-specific mRNA was markedly induced by the synthetic androgen mibolerone. Stimulation by two antiandrogens, CA and Flu-OH, was less pronounced. When the synthetic androgen and excess antiandrogen were applied simultaneously, the stimulation was reduced to the level seen with antiandrogens alone (Figure 3).

The expression of c-*myc* RNA and protein has been strongly implicated in the control of cell growth and differentiation (for review see Spencer & Groudine, 1990). In many cell types, inhibition of proliferation or induction of terminal differentiation is accompanied by down-regulation of c-*myc*. Therefore, we examined the effect of the synthetic androgen mibolerone on c-*myc* RNA expression in LNCaP cells. As shown in Figure 3, the steady-state level of c-*myc* RNA clearly decreases compared to a housekeeping mRNA. In the presence of excess antiandrogens, no change of c-*myc* RNA levels is detectable. C-*myc* expression and proliferative activity appear to be coupled in LNCaP cells. Work is in progress to determine the type of control governing the down-regulation of c-*myc* by mibolerone.

### *Inhibition of anchorage-independent growth*

Contact inhibition of cell proliferation and anchorage-independent growth are regarded as important parameters of the malignant phenotype of cells cultured *in vitro* (Freedman & Shin, 1974; Pollack *et al.*, 1984). Although in the presence of synthetic androgens, LNCaP cells constantly grew more slowly than control cells over prolonged periods of incubation, they did not show a clear-cut contact inhibition, as determined by long-term culture (data not shown). In contrast, when assayed for the ability of colony formation in soft agar, synthetic androgens drastically suppressed the growth potential of LNCaP cells (Figure 4). The number of colonies was reduced by a factor of between 50 to 500, i.e. to less than 1% of controls. The natural androgen DHT also inhibited anchorage-independent growth, but at $3.3 \times 10^{-9}$ M the colony number was still 39% of controls. A concentration of $3.3 \times 10^{-7}$ M of DHT was required to reduce the cloning

**Table II** Cell numbers (androgen receptor-negative cell lines PC-3, DU 145, and MRC-5; for comparison LNCaP) in mibolerone containing samples relative to untreated controls

| *Hormone concentration, $\times 10^{-9}$ M:* | *0* | *3.3* | *33* | *330* | *3300* |
|---|---|---|---|---|---|
| Cell line | | | | | |
| PC-3 | 100 | 91 (6.8) | 93 (11.3) | 93 (4.7) | 79 (6.2) |
| DU 145 | 100 | 108 (8.3) | 92 (11.2) | 107 (7.5) | 102 (7.6) |
| MRC-5 | 100 | 107 (9.3) | 100 (2.1) | 107 (6.7) | 108 (9.40 |
| LNCaP | 100 | 23 (0.9) | 29 (4.0) | 27 (3.4) | 30 (4.2) |

Values represent the cell number in hormone containing samples as per cent of the cell number of untreated controls which are defined as 100%. The standard deviation is given in brackets. Equal cell numbers were seeded into cell culture dishes, the medium was supplemented with the respective hormones, and after 120 h of incubation (192 h for MRC-5 cells) the cell numbers were determined manually. Each value represents the mean and standard deviation of at least six independent determinations.

**Figure 2** Synthetic androgens induce morphological changes in LNCaP cells. LNCaP cells were grown **a**, for 120 h without hormones, **b**, in the presence of $3.3 \times 10^{-9}$ M mibolerone, and **c**, in the presence of $3.3 \times 10^{-9}$ M mibolerone plus $1.8 \times 10^{-6}$ M CA. Magnification 560 ×; bar, 10 µm.

efficiency in soft agar to 0.6% of control cells (data not shown). Antiandrogens again counteracted the inhibition of anchorage-independent growth (Figure 4).

As shown in Figure 5, control LNCaP cells form large colonies in soft agar, while in the presence of mibolerone only occasional very small colonies are seen. When anti-androgen is applied simultaneously, the soft agar colonies reach about half the size as in control samples, and are less dense. The transformed behaviour *in vitro* is partially restored by CA and Flu-OH.

### Discussion

The androgen analogues were capable of inducing five distinct androgen effects (reduction of proliferation, inhibiton of anchorage-independent growth, change of morphology, induction of prostate specific mRNA and repression of c-*myc* RNA) on LNCaP cells to the full extent, a concentration of $3.3 \times 10^{-9}$ M being maximally effective. All growth-related effects were clearly antagonised by excess antiandrogens

**Figure 3** Mibolerone raises the level of PSA specific mRNA while repressing c-*myc* RNA. RNA was extracted as described after 48 h of incubation of LNCaP cells in the presence of the respective hormones. MIB, 1 ng ml$^{-1}$: mibolerone, $3.3 \times 10^{-9}$ M; CA, 750 ng ml$^{-1}$: cyproterone acetate, $1.8 \times 10^{-6}$ M; FLU-OH, 750 ng ml$^{-1}$: hydroxyflutamide, $2.6 \times 10^{-6}$ M. PGK, phosphoglycerate kinase.

**Figure 4** Synthetic androgens suppress colony formation of LNCaP cells in soft agar, and antiandrogens restore anchorage-independent growth. Colony numbers (means of at least 6 determinations) scored after 10–12 days of culture in hormone containing samples are represented as per cent of controls (*r*). For mibolerone, R1881, Sch B, and Sch C the value was less than one per cent and could not be accurately depicted. MIB, 1 ng ml$^{-1}$: mibolerone, $3.3 \times 10^{-9}$ M; Sch A, 1 ng ml$^{-1}$: 1α-17α-Dimethyl-testosterone, $3.3 \times 10^{-9}$ M; Sch B, 1 ng ml$^{-1}$: 1α-7α-17α-Trimethyl-testosterone, $3.3 \times 10^{-9}$ M; Sch C, 1 ng ml$^{-1}$: 7α-17α-Dimethyl-testosterone, $3.3 \times 10^{-9}$ M; CA, 750 ng ml$^{-1}$: cyproterone acetate, $1.8 \times 10^{-6}$ M; FLU-OH, 750 ng ml$^{-1}$: hydroxyflutamide, $2.6 \times 10^{-6}$ M.

which compete for androgen receptor binding. The identical dose-response behaviour suggests that all five phenomena are mediated by the same androgen receptor-dependent signal transducing mechanism.

The androgen receptor content of LNCaP cells appears to vary markedly among different sublines (Horoszewicz *et al.*, 1983: 153 and 266 fmol mg$^{-1}$ cytosolic protein; Sonnenschein *et al.*, 1989: 68 fmol mg$^{-1}$ cytosolic protein; Schuurmans *et al.*, 1988*c*: 920 fmol mg$^{-1}$ cytosolic protein), and even the existence of androgen-resistant sublines of LNCaP has been demonstrated (Hasenson *et al.*, 1985; van Steenbrugge *et al.*, 1989). The androgen receptor level of our stock of LNCaP cells was between 25 and 38 fmol mg$^{-1}$ cytosolic protein

**Figure 5** Mibolerone inhibits colony formation of LNCaP cells in soft agar. LNCaP cells were grown for 10 days in soft agar, **a**, without hormones, **b**, in the presence of $3.3 \times 10^{-9}$ M mibolerone, and **c**, in the presence of $3.3 \times 10^{-9}$ M mibolerone plus $1.8 \times 10^{-6}$ M CA. Magnification 100 ×; bar, 100 μm.

(H. Bojar, University of Düsseldorf, personal communication). Therefore we examined, whether the androgen-dependent signal transduction pathway in LNCaP cells could trigger induction of a prostate specific mRNA. The level of PSA mRNA was clearly elevated in the presence of $3.3 \times 10^{-9}$ M of the synthetic androgen mibolerone, and was also increased in the presence of high doses of antiandrogens ($1.8 \times 10^{-6}$ M CA or $2.6 \times 10^{-6}$ M Flu-OH; Figure 3). The simultaneous application of mibolerone and CA or Flu-OH did not result in synergistic enhancement of PSA transcription, but antiandrogens reduced androgen induction of PSA mRNA. Although both androgens and antiandrogens showed agonistic activity on PSA induction, they appear to compete for receptor binding and to cancel each other's activity.

Since the PSA promoter contains a consensus sequence of steroid receptor binding sites (Klobeck *et al.*, 1989), nuclear run-on experiments were conducted to show that enhanced transcription is indeed responsible for this hormone effect (D.A. Wolf, unpublished).

Recently, an amino acid change in the steroid binding domain of the LNCaP androgen receptor was found to result in an abnormallly high affinity of this receptor to CA and in stimulation of transcription by CA from a promoter containing a glucocorticoid responsive element (Veldscholte *et al.*, 1990*a*,*b*). The partial agonist activity of CA seen on PSA stimulation in LNCaP cells could be due to this mutation. However, the presence of the mutation in all sublines of LNCaP and its effect on androgen responsive elements remain to be established.

The concept of androgen dependence of normal and malignant prostatic cells has guided research and therapeutic strategies since the pioneering work of Huggins and Hodges (1941). Reduction of circulating testosterone from the normal range of 3–6 ng $ml^{-1}$ of serum ($= 1 \times 10^{-8}$ M–$2 \times 10^{-8}$ M) to about 0.3 ng $ml^{-1}$ ($= 1 \times 10^{-9}$ M) by surgical or to about 0.06 ng $ml^{-1}$ ($= 2 \times 10^{-10}$ M) by medical castration (Robinson & Thomas, 1971) leads to at least partial remission of androgen-dependent cancers. Growth properties of LNCaP cells characterised in this report and by others refute the hypothesis of androgen dependence of this cell line. LNCaP cells grow well in routine cell culture medium containing about 0.02 ng $ml^{-1}$ ($= 0.7 \times 10^{-10}$ M) testosterone and androstenedione, respectively (Challis *et al.*, 1974), i.e. far less than sera of orchiectomised men (Robinson & Thomas, 1971). Addition of 750 ng $ml^{-1}$ antiandrogen ($= 1.8 \times 10^{-6}$ M CA or $2.6 \times 10^{-6}$ M Flu-OH) to this medium only slightly affects cell proliferation (Figure 1). Addition of synthetic androgens at concentrations which maximally stimulate the androgen receptor (Veldscholte *et al.*, 1990*b*) does not lead to stimulation, but to inhibiton of cell growth (Table I). These results unequivocally demonstrate androgen receptor-mediated negative control of proliferation in LNCaP cells. All experimental data obtained with physiological androgen concentrations are consistent with inhibition of proliferation triggered by an androgen receptor-dependent mechanism. Since synthetic derivatives of steroid hormones can act as directly cytotoxic agents (Schulz *et al.*, 1988*a*), we examined, whether the inhibitory effect of synthetic androgens toward LNCaP cells is specific for this androgen receptor positive cell type. All control cell lines devoid of androgen receptors were in no way affected by the androgens tested. No indication of a cytotoxic mode of action was detected at the concentrations of the synthetic hormones used. The slight inhibition of LNCaP cells by 750 ng $ml^{-1}$ of antiandrogens may be due to a partial agonist effect at the androgen receptor, as shown by Veldscholte *et al.* (1990*b*) for CA. Since the neoplasm, from which the LNCaP line originated, had only poorly responded to androgen deprivation (Horoszewicz *et al.*, 1980), an androgen-dependent growth pattern of this cell line would have required a major change of behaviour during the nude mouse passages or the subsequent adaptation of the cells to *in vitro* growth.

The most salient phenomenon is the androgen receptor-mediated inhibition of cell proliferation and focus formation in soft agar, which suggests the suppression of the transformed phenotype. The finding of Sonnenschein *et al.* (1989) that LNCaP cells do not grow in castrated male nude mice implanted with a DHT pellet, is in accord with our result demonstrating androgen-induced inhibition of anchorage-independent growth of LNCaP cells *in vitro*. The effect of synthetic androgens on tumour formation of LNCaP cells in nude mice has not been investigated. In the vast majority of cell types inability to grow in soft agar correlates with drastically reduced tumourigenicity in an immunocompromised host (Pollack *et al.*, 1984; Freedman & Shin, 1974).

Induction of terminal differentiation of tumour cells *in vitro* is accompanied by loss of mitotic activity and of the transformed phenotype, and often involves a decrease in c-*myc* expression (for a review, see Spencer & Groudine, 1990, and Reiss *et al.*, 1986). The antiandrogens CA and Flu-OH did not exhibit partial agonist, i.e. repressing, activity on c-*myc* RNA levels. When androgens and antiandrogens were applied together, the androgen-induced repression of c-*myc* RNA was strongly antagonised. This behaviour is in contrast to the stimulation of PSA mRNA, where antiandrogens showed considerable androgenic activity.

Although no markers of differentiation in prostate epithelium are known, the induction of five fundamental changes in the behaviour of the cells (inhibition of proliferation; abrogation of anchorage-independent growth; morphological change; induction of Prostate Specific Antigen and repression of c-*myc*) may indicate the entry of the cells into a higher degree of differentiation. In some cell types at least, down-regulation of c-*myc* is supposed to have a functional role in the induction of differentiation, since the reduction of c-*myc* RNA and protein levels by antisense nucleic acids can trigger terminal differentiation in HL 60 cells (Holt *et al.*, 1988). It will be of interest to see, whether androgen-inducible growth controlling genes (Klein, 1987; Sager, 1989) are responsible for the unique reversion of malignant traits and induction of a more highly differentiated phenotype in LNCaP cells.

There are reports of so far inexplicable responses of prostatic carcinoma patients to administration of testosterone (Brendler *et al.*, 1950; Prout & Brewer, 1967; Crowin *et al.*, 1970). In rare cares, testosterone along led to objective improvement of the patient's condition. LNCaP could provide a useful *in vitro* model of an uncommon class of prostatic cancer which responds favourably to androgen administration and contribute to the understanding of the molecular mechanisms involved in the suppression of the transformed phenotype.

The expert technical assistance of Laura Chaudhuri and Dorothea Blaschke is gratefully acknowledged. We wish to thank Dr M. Töpert (Schering AG) for the gift of synthetic androgens, and Dr D. Eick for the c-*myc* probe and critical reading of the manuscript. This work was supported by Deutsche Forschungsgemeinschaft and Fonds der Chemischen Industrie.

### References

AUFFRAY, C. & ROUGEON, F. (1980). Purification of mouse immunoglobulin heavy-chain messenger RNAs from total myeloma tumor RNA. *Eur. J. Biochem.*, **107**, 303.

BERNS, E.M.J.J., DE BOER, W. & MULDER, E. (1986). Androgen dependent growth regulation of and the release of specific protein(s) by the androgen receptor containing human prostate tumor cell line LNCaP. *Prostate*, **9**, 247.

BONNE, C. & RAYNAUD, J.-P. (1976). Assay of androgen binding sites by exchange with methyltrienolone (R1881). *Steroids*, **27**, 497.

BRENDLER, H., CHASE, W.H. & SCOTT, W.W. (1950). Prostatic cancer: further investigation of hormonal relationships. *Arch. Surg.*, **61**, 433.

CHALLIS, J.R.G., KIM, C.K., NAFTOLIN, F., JUDD, H.L., YEN, S.S.C. & BENIRSCHKE, K. (1974). The concentration of androgens, oestrogens, progesterone and luteinizing hormone in the serum of foetal calves throughout the course of gestation. *J. Endocrinol.*, **60**, 107.

CORWIN, S.H., MALAMENT, M., SMALL, M. & STRAUSS, H.D. (1970). Experience with P-32 in advanced carcinoma of the prostate. *J. Urol.*, **104**, 745.

EICK, D., PIECHACZYK, M., HENGLEIN, B. & 6 others (1985). Aberrant c-*myc* RNAs of Burkitt's lymphoma cells have longer half-lives. *EMBO J.*, **4**, 3717.

FREEDMAN, V.H. & SHIN, S. (1974). Cellular tumorigencitiy in *nude* mice: correlation with cell growth in semi-solid medium. *Cell*, **3**, 355.

HASENSON, M., HARTLEY-ASP, B., KIHLFORS, C., LUNDIN, A., GUSTAFSSON, J.-Å. & POUSETTE, Å. (1985). Effect of hormones on growth and ATP content of a human prostatic carcinoma cell line, LNCaP-r. *Prostate*, **7**, 183.

HOLT, J.C., REDNER, R.L. & NIENHUIS, A.W. (1988). An oligomer complementary to c-*myc* RNA inhibits proliferation of HL-60 promyelocytic cells and induces differentiation. *Mol. Cell. Biol.*, **8**, 963.

HOROSZEWICZ, S.J., LEONG, S.S., MING CHU, T. & 8 others (1980). The LNCaP cell line – a new model for studies on human prostatic carcinoma. *Prog. Clin. Biol. Res.*, **37**, 115.

HOROSZEWICZ, S.J., LEONG, S.S., KAWINSKI, E. & 5 others (1983). LNCaP model of human prostatic cancer. *Cancer Res.*, **43**, 1809.

HUGGINS, C. & HODGES, C.V. (1941). Studies on prostatic cancer. 1. The effect of castration, of estrogen and of androgen injection on serum phosphatases in metastatic carcinoma of the prostate. *Cancer Res.*, **1**, 293.

KAIGHN, M.E., NARAYAN, K.S., OHNUKI, Y., LECHNER, J.F. & JONES, L.E. (1979). Establishment and characterization of a human prostatic carcinoma cell line (PC-3). *Invest. Urol.*, **17**, 16.

KLEIN, G. (1987). The approaching era of the tumor suppressor genes. *Science*, **238**, 1539.

KLOBECK, H.-G., COMBRIATO, G., SCHULZ, P., ARBUSOW, V. & FITTLER, F. (1989). Genomic sequence of human prostate specific antigen (PSA). *Nucleic Acids Res.*, **17**, 3981.

LIPPMAN, S.M., KESSLER, J.F. & MEYSKENS, F.L. Jr (1987). Retinoids as preventive and therapeutic anticancer agents. *Cancer Treat. Rep.*, **71**, 391 and 493.

MICHELSON, A.M., MARKHAM, A.F. & ORKIN, S.H. (1983). Isolation and DNA sequence of a full-length cDNA clone for human X chromosome-encoded phosphoglycerate kinase. *Proc. Natl Acad. Sci. USA*, **80**, 472.

POLLACK, R., CHEN, S., POWERS, S. & VERDERAME, M. (1984). Transformation mechanisms at the cellular level. In *Advances in Viral Oncology*, Vol. 4, Klein, G. (ed.) pp. 3–28. Raven Press: New York.

PROUT, G.R. & BREWER, W.R. (1967). Response of men with advanced prostatic carcinoma to exogenous administration of testosterone. *Cancer*, **20**, 1871.

RAYNOR, R.H., HAZRA, T.A., MONCURE, C.W. & MOHANAKUMAR, T. (1984). Characterization of a monoclonal antibody, KR-P8, that detects a new prostate specific marker. *J. Natl Cancer Inst.*, **73**, 617.

REISS, M., GAMBA-VITALO, C. & SARTORELLI, A.C. (1986). Induction of tumor cell differentiation as a therapeutic approach: preclinical models for hematopoietic and solid neoplasms. *Cancer Treat. Rep.*, **70**, 201.

ROBINSON, M.R.G. & THOMAS, B.S. (1971). Effect of hormonal therapy on plasma testosterone levels in prostate carcinoma. *Br. Med. J.*, **4**, 391.

SAGER, R. (1989). Tumor suppressor genes: the puzzle and the promise. *Science*, **246**, 1406.

SCHULZ, P., BAUER, H.W. & FITTLER, F. (1985). Steroid hormone regulation of prostatic acid phosphatase expression in cultured human prostatic carcinoma cells. *Hoppe-Seyler's Z. Physiol. Chem.*, **366**, 1033.

SCHULZ, P., BAUER, H.W., BRADE, W.P., KELLER, A. & FITTLER, F. (1988*a*). Evaluation of the cytotoxic activity of diethylstilbestrol and its mono- and diphosphate towards prostatic carcinoma cells. *Cancer Res.*, **48**, 2867.

SCHULZ, P., STUCKA, R., FELDMANN, H., COMBRIATO, G., KLOBECK, H.-G. & FITTLER, F. (1988*b*). Sequence of a cDNA encompassing the complete mature human Prostate Specific Antigen (PSA) and an unspliced leader sequence. *Nucleic Acids Res.*, **16**, 6226.

SCHUURMANS, A.L., BOLT, J. & MULDER, E. (1988*a*). Androgens and transforming growth factor beta modulate the growth response to epidermal growth factor in human prostatic tumor cells (LNCaP). *Mol. Cell. Endocrinol.*, **60**, 101.

SCHUURMANS, A.L., BOLT, J. & MULDER, E. (1988*b*). Androgens stimulate both growth rate and epidermal growth factor receptor activity of the human prostate tumor cell line LNCaP. *Prostate*, **12**, 55.

SCHUURMANS, A.L., BOLT, J., VOORHORST, M.M., BLANKENSTEIN, R.A. & MULDER, E. (1988*c*). Regulation of growth and epidermal growth factor receptor levels of LNCaP prostate tumor cells by different steroids. *Int. J. Cancer*, **42**, 917.

SCHUURMANS, A.L., BOLT, J. & MULDER, E. (1989). Androgen receptor-mediated growth and epidermal growth factor receptor induction in the human prostate cell line LNCaP. *Urol. Int.*, **44**, 71.

SONNENSCHEIN, C., OLEA, N., PASANEN, M.E. & SOTO, A.M. (1989). Negative controls of cell proliferation: human prostate cancer cells and androgens. *Cancer Res.*, **49**, 3474.

SPENCER, C.A. & GROUDINE, M. (1990). Control of c-*myc* regulation in normal and neoplastic cells. *Adv. Cancer Res.*, **55**, (in press).

STONE, K.R., MICKEY, D.D., WUNDERLI, H., MICKEY, G.H. & PAULSON, D.F. (1978). Isolation of a human prostate carcinoma cell line (DU 145). *Int. J. Cancer*, **21**, 274.

VAN STEENBRUGGE, G.J., GROEN, M., VAN DONGEN, J.W. & 5 others (1989). The human prostatic carcinoma cell line LNCaP and its derivatives. An overview. *Urol. Res.*, **17**, 71.

VELDSCHOLTE, J., VOORHORST-OGINK, M.M., BOLT-DE-VRIES, J., VAN ROOIJ, H.C.J., TRAPMAN, J. & MULDER, E. (1990*a*). Unusual specificity of the androgen receptor in the human prostate tumor cell line LNCaP: high affinity for progestagenic and estrogenic steroids. *Biochim. Biophys. Acta*, **1052**, 187.

VELDSCHOLTE, J., RIS-STAPLERS, C., KUIPER, G.G.J.M. & 7 others (1990*b*). A mutation in the ligand binding domain of the androgen receptor of human LNCaP cells effects steroid binding characteristics and response to antiandrogens. *Biochem. Biophys. Res. Commun.*, **173**, 534.

WAKELING, A.E., FURR, B.J.A., GLEN, A.T. & HUGHES, L.R. (1981). Receptor binding and biological activity of steroidal and non-steroidal antiandrogens. *J. Steroid Biochem.*, **15**, 355.

WAXMAN, S., ROSSI, G.B. & TAKAKU, F. (eds) (1988). *The Status of Differentiation Therapy of Cancer*. Serono Symposia Publications from Raven Press, Vol. 45: New York.

WILDING, G., CHEN, M. & GELMANN, E.P. (1989). Aberrant response *in vitro* and hormone-responsive prostate cancer cells to antiandrogens. *Prostate*, **14**, 103.